# On the Use of Argentum Nitratum in Ulcer

**Published:** 1808-02

**Authors:** 


### Medical and Phyſical Journal.

VOL. XIX.] FEBRUARY, 1808. [NO. 108.

*Printed for R. PHILLIPS, by W. Thorne, Red Lion Court, Fleet Street, London*

### *To the Editors of the Medical and Physical Journal.*

GENTLEMEN,

WHEN an injury is done to the upper part of the great toe, in consequence of a heavy weight falling on it, or of its being subjected to severe pressure, a sore is frequently occasioned on that part of the toe where the nail grows; the nail itself comes away, either entirely, or in part, and a very ugly, ill-conditioned ulcer torments the patient; the edges become hard and raised; the surface is of a brownish red colour, instead of a healthy florid red; it has a glassy appearance, and the discharge from it is thin and serous. The patient is rendered lame, and sometimes suffers a good deal of pain and uneasiness from the sore. Having observed the trouble which such ulcers give to the patient, and indeed not unfrequently to the surgeon, who in the usual mode of treatment finds them extremely difficult to heal, I am induced to send you this letter for the purpose of describing two cases, where the mode of practice I adopted perfectly succeeded, and that in a short space of time. That you may be satisfied as to the authenticity of the communication, I enclose you my address; but for reasons, which it is needless here to mention, request that only my initials may be affixed to the letter when printed.

Some years ago, while I was dresser to a surgeon of one of the hospitals in the metropolis, a girl, I think about 13 years old, came into the hospital, and was placed under his care. She had on one of her great toes a sore of the above description. I do not know precisely how long this sore had existed; but I well know, that it was by no means of a recent date. It occupied the usual place, I mean the part where the nail (which now was entirely gone) had been situated; and exactly answered the description given above with respect to its edges, discharge, and colour. The pain from it was not considerable; the size not quite so large as the nail must originally have been. All the means usually had recourse to on these occasions, poultices, salves, washes, &c. of different kinds were employed, but without effect: the sore continued nearly stationary. At length, the surgeon, as a last resource, and to save the patient from the trouble of a constant open sore, proposed to amputate the first joint of the toe. I begged, that, before the operation was performed, he would permit me to try one more remedy, to which assent was most readily given. I then scraped some argentum nitratum, and heaped it up over the ulcer, which formed a cup capable of receiving a pretty large quantity. The discharge from the toe itself moistened the caustic, so that it soon produced very considerable pain. A kind of brownish white membrane was formed over the sore. The whole was afterwards covered with a rag dipped in a wash composed of aqua calcis and calomel. At this distance of time I cannot call to mind how often the caustic was applied; but I well recollect, that no other applications were used besides the above, and that the toe was quite well in two weeks from the period of the first application.

A considerable time afterwards, a man came to me with a similar sore, the particulars of which I am capable of detailing with a much greater degree of accuracy. He was about 50 years of age, and acted in the capacity of a watchman, at the brewery in Weston-street, Southwark; so that he was almost constantly on his legs during the night. He likewise used exercise during part of the day; I believe, as a porter at the same place: during the rest of the day he was in bed. He told me, that a horse had trod on his foot about 18 months before I saw him, and had bruised the upper part of his toe. He had applied to various medical men; but had not experienced any relief from their attentions. In this instance the nail of the toe was not completely gone; but still perhaps about a third part of it remained. The pain which he experienced was constant, and tormented him considerably. The other appearances nearly answered the description given above. Having been always accustomed in these cases to consider the nail as an irritating cause, I doubted much, whether the toe would get well till the nail came away completely. However, as my treatment of the former case had been so successful, I determined to use the canstic again in this instance. I likewise recommended rest to the man: but, with with this injunction, he told me he was totally unable to comply, as he had a wife and family entirely dependant on his labour. I therefore applied a large quantity of powdered caustic to his toe in the morning just before his bed-time, and covered the toe with some simple ointment spread on rag. It did not at the moment of application occasion him much pain; but in a few minutes the pain became most excessively severe, so that, although he lived only about a quarter of a mile from me, he thought he should scarcely be able to reach his home. He then lay down in his bed. The pain continued excessively severe during 6 hours, and quite deprived him of rest. It then went off. In about 3 days, when he again visited me, he said that the toe had continued perfectly easy from that time to the moment of my seeing him. On inspecting the ulcer, there appeared over it the brownish white membrane. The thick edges round the sore were burnt by the caustic, as was also some of the nail. I cut away part of the burnt nail, and with the forceps tore off the membrane over the sore, and the thick skin around it, which, in consequence of its being destroyed by the caustic, was removed without much difficulty. The ulcer had now a much more healthy appearance; and the skin under that which was torn away appeared thin, and much redder than before. I again applied the caustic, and gave my patient 2 grains of opium to take as soon as he reached his home. When he repeated his visit to me in 3 or 4 days, the sore appeared still more healthy. The pain arising from the application of the caustic, although by no means inconsiderable, was not so severe as it had been before. I think it was at this third dressing I applied adhesive plaster firmly round the toe. I afterwards continued to dress it about twice a week, exactly in the same manner, excepting that towards the end of the treatment I decreased the quantity of caustic. As the toe got better, the pain occasioned by the use of this remedy still diminished. By this management, in three weeks from the first application of the argentum nitratum, the ulcer was completely healed. A part of the nail still remained, the skin having shot from underneath it, to meet that which was coming from the end and sides of the toe.

It might perhaps be imagined, that a more gentle application of caustic would prove equally effectual, and not occasion so much pain to the patient. It is true, it would not give him at any one time so much pain; but I believe would not be by any means so effectual. I believe this not

 merely merely from theory, but from a case which I saw. In this instance I recommended the free use of the caustic to the practitioner who had the care of the patient. He did use the caustic; but contented himself with touching the ulcer with it. This, however, did not seem to me to quicken the cure of the part, as caustic used according to the statement given above has done.

The application of caustic probably contributes in more ways than one to the healing of this ulcer. Its surface was in a sluggish indolent state, as was sufficiently evident from the age of the ulcer, its brown appearance, and the serous nature of the discharge. The caustic, in this large quantity, compleatly eat away the upper diseased surface of the sore; and, by chemically uniting itself with what it had thus acted on, formed, what appeared to be, the brownish membrane. But this was not all: the part immediately below, which was not destroyed by the caustic, must have been considerably stimulated by it; and thus inclined to act with a much greater degree of vigour than before its application. The skin likewise round the ulcer was as indolent as the surface of the sore itself; so that it was not disposed to shoot out the vessels, which appear to be necessary, in order to the formation of new skin over the wound; but the edges of the surrounding skin were acted on exactly as the surface of the sore. The diseased portions close to the ulcer were compleatly eaten away: and the edge now nearest the ulcer stimulated to act with much more vigour than before. The surrounding skin, also, by being much thickened, would not contract with nearly so much ease as when, from the application of the caustic, it was rendered thinner. The adhesive plaster in the last case, might perhaps contribute something to the healing of the ulcer, by supporting the parts, and bringing the edges of the ulcer into closer contact. But, that the principal cause of this cure was to be attributed to the caustic, is evident from the first case, in which no adhesive plaster was used; and yet the ulcer was healed in a smaller space of time than that in the last case.

I am, &c.

J. B.

*London, December* 21, 1807.

